# Nucleated Red Blood Cells as a Marker of Acute and Chronic Fetal Hypoxia in a Rat Model

**DOI:** 10.5041/RMMJ.10302

**Published:** 2017-04-28

**Authors:** Victoria K. Minior, Brian Levine, Asaf Ferber, Seth Guller, Michael Y. Divon

**Affiliations:** 1 Department of Obstetrics & Gynecology, Lenox Hill Hospital, New York, New York, USA; 2 Colorado Center for Reproductive Medicine, New York, New York, USA; 3 Department of Obstetrics & Gynecology, Yale University, New Haven, Connecticut, USA

**Keywords:** Fetal growth restriction, fetal hypoxia, nucleated red blood cells

## Abstract

**Objective:**

To examine the relationship between duration of fetal hypoxia, nucleated red blood cell (NRBC) count, and fetal growth.

**Methods:**

Pregnant rats were exposed to a severe hypoxia (9.5%–10% O_2_) for varying time intervals (2, 6, 12, 24, 48, and 120 hours; *n*=4 for each time interval) immediately prior to delivery at term. Normoxic controls were exposed to room air (21% O_2_) and matched for all other study variables (*n*=4 rats for each time interval). Pups were delivered via hysterotomy while maintaining exposure gas concentrations. Blood gas analysis and NRBC counts were performed, and fetal body and liver weights were recorded. Student’s *t* test and simple regression were used for statistical analysis.

**Results:**

As the duration of hypoxia increased, fetal weight, liver weight, blood bicarbonate, and base excess levels decreased significantly; concomitantly, NRBC counts increased. This increase in NRBCs became statistically significant after 24 hours of exposure. After 48 hours of hypoxia there was a 2.5-fold rise in NRBC count, and after 120 hours of hypoxia there was a 4.5-fold rise in NRBC count over control levels. After 12 or more hours of hypoxia, fetal body weights were significantly reduced; 120 hours of hypoxia resulted in a 35% reduction in fetal body weight, a 34% reduction in fetal liver weight, and 356% increase in NRBC count.

**Conclusion:**

In a pregnant rat model, chronic maternal hypoxia (≥24 hours) results in a significant increase in fetal NRBC counts as well as reduced fetal body weight and organ growth.

## INTRODUCTION

The presence of elevated levels of nucleated red blood cells (NRBCs) in the peripheral blood of newborns has been traditionally attributed to “asphyxial conditions.”^1^–^3^ Increased numbers of these cells have been demonstrated in association with acidemia at birth^4^,^5^ and conditions associated with “placental insufficiency” such as fetal growth restriction^6^–^9^ as well as “intrapartum fetal distress.”^10^,^11^ Furthermore, elevated NRBCs have been noted in the newborns of mothers who smoke cigarettes and mothers exposed to excessive air pollution.^12^,^13^ A direct correlation between decreasing umbilical artery pH values and NRBC elevation has been noted in the term human fetus at birth.^4^,^5^ Similar findings were reported by Soothill et al. studied umbilical cord blood samples attained through cordocentesis from growth-restricted fetuses and demonstrated that the severity of both fetal hypoxemia and fetal acidemia directly correlated with the degree of NRBC elevation.^14^

Human newborns with elevated levels of NRBCs at birth have been shown to suffer a significant increase in both short-term morbidity and mortality and long-term disability. Neonatal complications such as severe respiratory distress, circulatory instability,^7^,^8^ necrotizing enterocolitis,^15^ retinopathy of prematurity,^16^ hypoxic-ischemic encephalopathy,^17^ early-onset neonatal seizures,^18^ prolonged neonatal intensive care unit stay, and neonatal demise^10^ have been reported in association with elevated NRBCs at birth. In addition, significant associations between elevated NRBCs at birth and lifelong complications such as developmental delay^17^ and cerebral palsy^1^,^19^,^20^ have been described.

Several authors have speculated that fetal NRBC counts represent a useful marker of chronic intrauterine hypoxia resulting in neurologic injury.^1^,^4^,^20^ Although this marker may have significant potential utility in the medico-legal setting, it seems premature to make this suggestion without scientific documentation of cause and effect.^18^ In fact, to date, the effect of duration of fetal hypoxia on elevation of NRBCs has not been adequately studied. Because of the inability to perform a controlled investigation of the effect of hypoxia on NRBC counts in the human fetus, a pregnant rat model was used in the present study to examine the dose–response effect of acute and chronic fetal hypoxia on fetal blood gas parameters, NRBC count, and birth weight.

## MATERIAL AND METHODS

### Animals

This investigation was approved by the New York University Institutional Animal Care and Use Committee, and all animal care was in strict accordance with National Institutes of Health guidelines. Young adult, nulliparous, Sprague-Dawley rats were purchased from Charles River Laboratories (Raleigh, NC, USA) and were housed in the Division of Laboratory Animal Research facility at the New York University Medical Center. Rats were individually maintained in polycarbonate cages in an environmentally controlled vivarium under a 12-hour-light-12-hour-dark cycle. They received a standard laboratory rodent diet. Free access to food and water was provided throughout the experiment. To ensure accurate gestational age dating of pregnancy, rats were mated at our facility. Male and female rats were housed together overnight and mating was confirmed by the presence of a vaginal plug the following morning (day 0 of gestation).

### Experimental Design

Pregnant rats were randomly allocated to exposure type (hypoxia or control groups) and duration (2, 6, 12, 24, 48, or 120 hours). Four pregnant rats were included in each group, for each exposure duration. Thus, a total of 48 rats were studied. Pregnant rats in their polycarbonate cages were placed in a specially designed, normobaric, Plexiglas chamber (15×13×22.5 inches; 38.1×33×57.1 cm) for the designated exposure preceding delivery. Delivery by hysterotomy occurred on day 21 of gestation (term) for all rats. While study rats were in the chamber, a severely hypoxic environment was created by titrating compressed air and nitrogen to produce the desired oxygen concentration of 9.5%–10%. Previous investigators have shown that fetal wastage occurs when the inspired fraction of oxygen falls below 9.5%.^21^ Carbon dioxide, humidity, and ammonia were eliminated from the chamber by maintaining continuous flow of air through the chamber at a rate of 1.0 L/minute. Dual stage cylinder regulators (TW Smith, Brooklyn, NY, USA) connected to the gas tanks controlled the flow of gas into a bench top flow meter/mixer-proportioner (Matheson Tri-gas, Montgomeryville, PA, USA) positioned between the Plexiglas chamber and the gas tanks. Prior to initiation of a hypoxic exposure, the oxygen concentration in the Plexiglas chamber was gradually lowered to 10% via 1.5 L/minute compressed nitrogen infusion over 1 hour. Control rats were exposed to 21% oxygen (room air) and matched for all other study variables. Oxygen concentration was continuously monitored by an oxygen analyzer (Hudson RCI, Temecula, CA, USA).

At the completion of the exposure period, rats were immediately placed in an isoflurane chamber and anesthetized. During the period of anesthesia, the original exposure gas concentrations were maintained. Anesthesia was maintained throughout the surgery using a rodent nose cone anesthesia machine with 2.5% isoflurane. Once adequate anesthesia was achieved, laparotomy was performed and the first uterine horn encountered was exteriorized. An incision was made in the uterine horn opposite the placental insertion site to expose each pup, with the lowermost pup sampled first. Arterial blood gas measurements as well as blood smears were immediately collected from the first six pups occupying the base of the uterine horn and extending cephalad on one side. Blood gases were sampled using the technique of Lueder et al.,^22^ where the fetal axillary artery was isolated and severed, and blood was collected into a capillary tube. Throughout this time, the placental circulation remained intact. Blood gas analysis was performed using a Radiometer 700 (Radiometer Medical ApS, Brønshøj, Denmark) series blood gas analyzer on 55-μL samples. Subsequently, the mother rat was euthanized with a lethal dose of pentobarbital. Pup weights and location within the uterine horn were documented, and pups were euthanized with a lethal injection of pentobarbital. Subsequently, the fetal liver was dissected and weighed.

### Nucleated Red Blood Cell Counts

Peripheral blood smears were stained with Wright stain. Nucleated red blood cell (NRBC) counts were obtained with the use of a manual cell counter per 100 white blood cells (WBCs) by two independent observers blinded to both exposure type and duration. Nucleated red blood cell counts were obtained for the first six pups delivered, extending from the lowermost to mid uterine horn from each pregnancy. The mean NRBC count for each pregnancy was then used in the data analysis.

### Data Analysis

Statistical analysis was performed using Statview (version 5.0, SAS technologies) and SPSS (version 13.0). Statistical analysis included Student’s *t* test and simple regression where appropriate. Statistical comparisons were made between mean values for each pregnancy for each group unless otherwise specified. For blood gas analysis, only the first blood gas attained was used for statistical comparisons for each pregnancy because results obtained from subsequent pups had an increased potential to be influenced by the physiologic effects of inhaled maternal anesthesia. *P<*0.05 was considered statistically significant.

To assess repeatability of NRBC counts, the intraclass correlation coefficient (ICC) was calculated using the estimates of within- and between-observer variability obtained from a two-way mixed effects analysis of variance model. The ICC assesses the agreement of quantitative measurements in terms of consistency and conformity. The ICC ranges from 0 to 1, where 1 demonstrates perfect reliability and a value of 0 indicates no repeatability of the measurement. The ICC is commonly interpreted as follows: ICC<0.4 indicates poor repeatability; 0.4<ICC<0.75 indicates fair to good repeatability; and ICC>0.75 indicates good to excellent repeatability.^23^

## RESULTS

All pups delivered were viable (*n*=669). There were no spontaneous preterm or term deliveries. Two of the four gestations exposed to 48 hours of hypoxia were associated with meconium staining of amniotic fluid in multiple sacs. The mean number of pups delivered per gestation was 14±2 (±SD). The number of pups per gestation was not significantly different between hypoxic and control rats at any time point.

### Fetal Blood Gas Values

**Figure 1:**
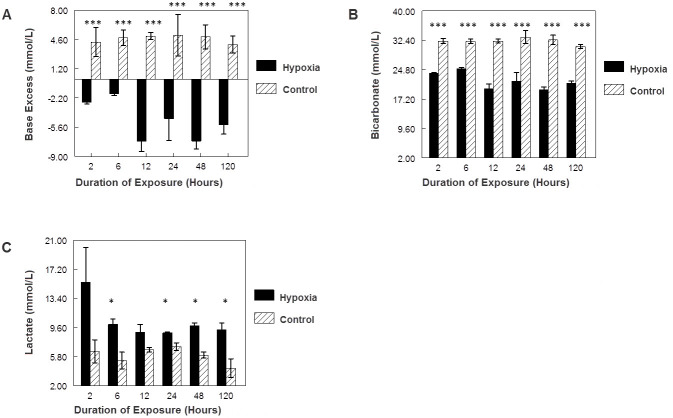
The Effect of Maternal Exposure to 10% Oxygen on Fetal Blood Gas Parameters. Rats exposed to hypoxia are represented by the dark bars, control rats are represented by the hatched bars, and error bars represent standard error of the mean. **(A)** Exposure duration versus base excess (***P<0.001 for all comparisons). **(B)** Exposure duration versus bicarbonate (***P<0.001 for all comparisons). **(C)** Exposure duration versus lactate (*P<0.05).

Maternal exposure to 9.5%–10% oxygen resulted in abnormal blood gas parameters in all hypoxia study groups. Study pups had significantly lower base excess (Figure 1A) and blood bicarbonate levels (Figure 1B) at all time points when compared to control pups. In addition, significantly higher blood lactate levels (consistent with metabolic acidosis) were also noted at 6, 24, 48, and 120 hour time points in the hypoxia group as compared to controls (Figure 1C).

### Nucleated Red Blood Cell Counts

**Figure 2:**
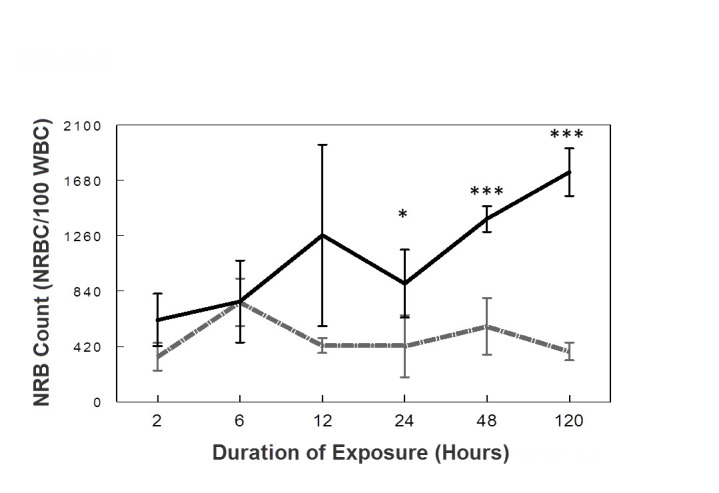
The Effect of Exposure Duration on Fetal NRBC Count. Control rats are represented by the grey line, study rats are represented by the black line, and error bars represent standard error of the mean. Statistically significant differences were noted between control and hypoxic gestations at 24, 48, and 120 hours (*P<0.05, ***P<0.001).

Maternal exposure to a severely hypoxic environment resulted in progressive elevations in fetal NRBC counts relative to controls. Statistically significant differences were noted between control and hypoxic gestations at 24, 48, and 120 hours (Table 1, Figure 2). After 48 hours of hypoxia, there was a 143% rise in NRBC count over control values (1388± 98 versus 572±214 NRBC/100 WBC, *P=*0.0005). After 120 hours of hypoxia, there was a 356% increase in NRBC count over control levels (1743±180 versus 382±67 NRBC/100 WBC, *P=*0.0004). A wide range in the NRBC count was noted in study and control rats at all time points (Table 1). However, at 48 hours, 88% of pups in the hypoxia group had an individual NRBC count above the normal range for controls, and at 120 hours 100% of pups in the hypoxia group had an individual NRBC count above the normal range for controls.

**Table 1:** With Increasing Duration of Hypoxia, There are Elevations in NRBC Counts over Control Levels.

Exposure Duration (hours)	Control Mean NRBC/100 WBC		Hypoxic Mean NRBC/100 WBC		*P* Value
	*n*±SEM	Range	*n*±SEM	Range	
2	342±104	230–473	621±199	351–774	0.05
6	756±180	496–901	761±311	358–1039	0.97
12	426±57	385–510	1264±688	657–2192	0.05
24	422±235	125–700	896±258	542–1138	0.03
48	572±214	415–874	1388±98	1304–1523	0.0005
120	382±67	205–523	1743±180	1427–2190	0.0004

## FETAL BIRTH WEIGHT

**Figure 3:**
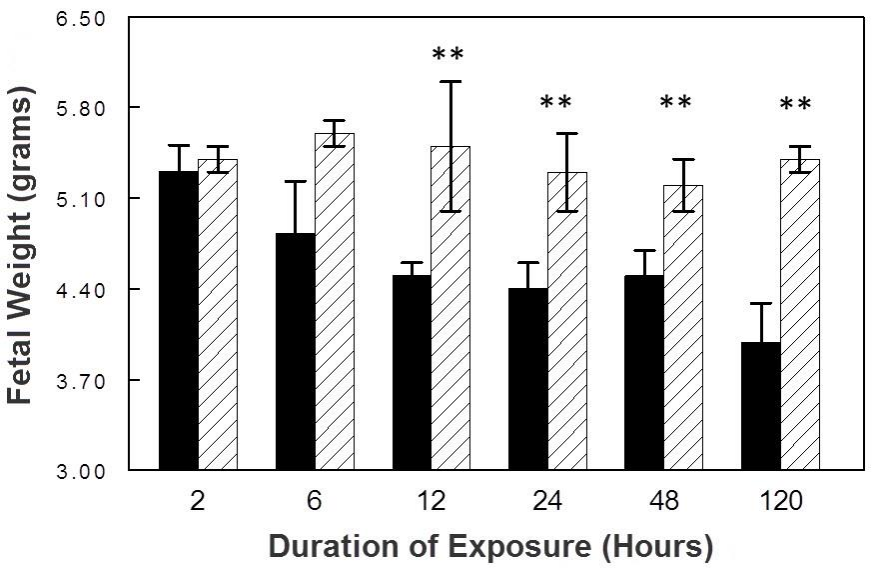
The Effect of Exposure Duration on Fetal Weight. Hypoxic rats are represented by the black bars, and control rats are represented by the hatched bars (**P<0.01).

The mean birth weight of control pups at term was 5.47±0.08 g. In the control group, there was no difference in birth weight with increasing durations of exposure to room air (Figure 3). As expected, prolonged maternal exposure to 9.5%–10% oxygen resulted in fetal growth restriction. As shown in Figure 3, as exposure duration increased, there were progressive decreases in fetal body weight in hypoxic rat pups relative to control rat pups. The difference between control and hypoxic fetal body weights reached statistical significance after 12 hours of hypoxia. After 120 hours of hypoxia, there was a 35% decrement in fetal weight relative to controls (3.99±0.3 g versus 5.39±0.09 g, *P=*0.004). In addition, there were progressive reductions in fetal liver weight with increasing durations of hypoxia. After 120 hours of hypoxia, there was a 34% decrement in mean fetal liver weight in study rats relative to controls (0.23±0.04 g versus 0.31±0.02 g, *P=*0.01).

### Correlations between NRBC Counts, Fetal Blood Gas Values, and Fetal Birth Weight

**Figure 4:**
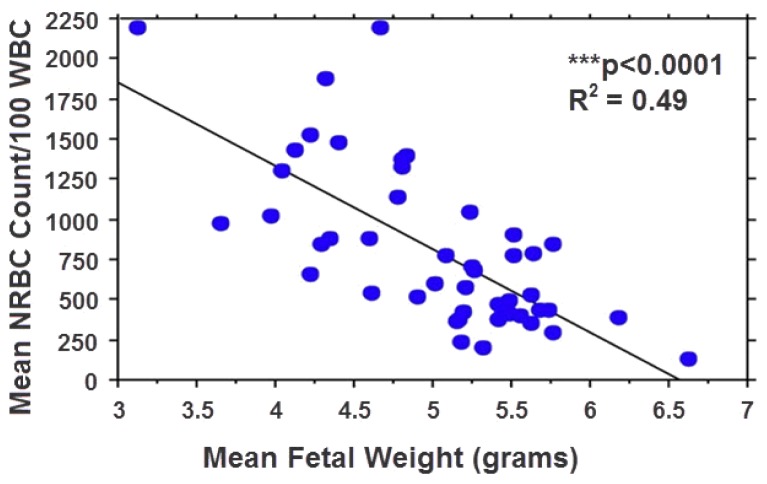
Relationship between NRBC Count and Fetal Weight. A significant negative correlation was noted between NRBC count and fetal weight (*R^2^*=0.49, ***P<0.0001).

Regression analysis revealed a significant correlation between NRBC counts and fetal metabolic acidosis as measured by fetal base excess (*R^2^*=0.46, *P<*0.0001), bicarbonate (*R^2^*=0.46, *P<*0.0001), and lactate (*R^2^*=0.18, *P=*0.005), indicating that NRBC is correlated with hypoxia. Moreover, a significant negative correlation was noted between fetal NRBC counts and fetal weight (*R^2^*=0.49, *P<*0.0001), with the smallest fetuses having the highest NRBC counts and the largest fetuses having the lowest NRBC counts (Figure 4), consistent with the observation of increase of NRBCs in human fetal growth restriction.^14^ In addition, simple regression analysis revealed a significant correlation between fetal body weight and the degree of metabolic acidosis as measured by fetal base excess (*R^2^*=0.5, *P<*0.0001), bicarbonate (*R^2^*=0.54, *P<*0.0001), and lactate (*R^2^*= 0.22, *P=*0.002).

Statistical evaluation of within-observer reliability revealed intraclass correlation coefficients of 0.95 (95% CI 0.678–0.993, *P=*0.001) and 0.98 (95% CI 0.88–0.998, *P<*0.0005). The ICC for interobserver variability was 0.8 (95% CI 0.540–0.904, *P<*0.0005). These results indicate good agreement within and between observers.

## DISCUSSION

This study demonstrates that, in the term rat fetus, increasing durations of fetal hypoxia result in significant and progressive elevations of NRBCs simultaneous with reductions in fetal body and organ growth. The degree of NRBC count elevation was related to the duration of intrauterine hypoxia. A statistically significant difference in NRBC count in study animals over controls was detected after 24 hours of hypoxia.

As normal human pregnancy advances, the number of circulating erythroblasts or NRBCs decreases exponentially, reaching relatively low levels by 26–28 weeks’ gestation.^24^ At this time, bone cavities become more functional, and a transition occurs from splenic and hepatic erythropoiesis, to medullary erythropoiesis. Prior to medullary erythropoiesis, erythroblasts freely circulate in the peripheral blood. As bone marrow erythropoiesis increases, erythroblasts become confined to the medullary parenchyma.^25^ In a normal human newborn delivered at term, there are very few nucleated erythrocytes in the peripheral blood. The mean number of NRBCs is 8.6 per 100 white blood cells, with a standard deviation of 10.3.^26^

The mechanism by which NRBCs become present in elevated numbers in the peripheral fetal circulation has been a topic of much debate. Animal and human fetuses that are delivered from a low-oxygen intrauterine environment have been noted to have an abundance of NRBCs in their peripheral blood.^2^,^27^ It is speculated that, in an effort to compensate for the lack of adequate oxygen supply, the mammalian fetus accelerates the erythropoietic process. This results in increased numbers of immature erythroblasts and thereby a potential improvement in fetal oxygen-carrying capacity. Erythropoietin is well known to be the primary hormone mediating erythropoiesis.^28^,^29^ Its secretion results in an increase in red blood cell mass by stimulating proliferation, differentiation, and maturation of erythroid precursors.^30^ In studies of fetal sheep, Widness et al. found that erythropoietin is elevated in response to intrauterine hypoxia approximately 4–6 hours after initiation of an hypoxic insult.^31^ Similar time responses have been documented by *in vivo* studies of adult rats.^32^ Increases in the number of circulating NRBCs can also occur as a result of hormone-mediated release of NRBCs from bone marrow stores.^33^ Erythropoietin has been shown to increase blood flow through bone marrow as well as increasing bone marrow porosity, allowing escape of the relatively large and rigid NRBCs into the peripheral circulation.^33^–^35^ In cases of long-standing, chronic hypoxia, increased extramedullary hematopoiesis has been demonstrated in animal models and in humans.^36^,^37^ As NRBCs freely circulate under these conditions, elevated peripheral blood NRBCs would be noted.

Acute elevations in peripheral NRBCs may occur secondary to an interleukin (IL)-mediated response.^10^ Recent *in vitro* studies have demonstrated that erythroid progenitor differentiation and maturation can be induced by IL-6 in the absence of added erythropoietin^38^,^39^ and that IL-6 is markedly increased in response to hypoxia in both *in vitro* studies and *in vivo* human studies.^40^,^41^

Unfortunately, animal studies regarding the association of NRBCs to fetal hypoxia have been few and inconclusive. Blackwell et al. exposed term pregnant rats to acute 2-hour periods of severe hypoxia and demonstrated that fetal NRBCs became significantly elevated 12–24 hours after the exposure. This transient elevation disappeared 36 hours later.^42^ Despite differences in study design, our results are consistent with the observations of Blackwell et al. in that we noted significant elevations in NRBC count at ≥24 hours after initiation of the exposure. However, our results lie in direct contrast to those of Ravishankar et al. who exposed pregnant term rats to 120 hours of severe hypoxia and concluded that chronic hypoxia did not result in significant elevations of NRBCs in the fetal circulation.^27^ These authors did, however, show that fetal NRBCs were significantly elevated following exposure to chronic hypoxia combined with nitric oxide inhibition, sufficient to produce fetal growth restriction.^27^ Unlike the results presented by Ravishankar et al., our results demonstrate a large and significant increase in NRBCs following 120 hours of continuous maternal exposure to severe hypoxia. Neither the Ravishankar et al. nor the Blackwell et al. studies were designed specifically to evaluate the impact of duration of fetal hypoxia on NRBC counts. This is particularly relevant as a multitude of human and animal studies have suggested that chronic fetal hypoxia has a profound impact on neurologic outcome. Our results indicate that 24 hours of continuous exposure to severe hypoxia is sufficient to result in a significant elevation in fetal NRBCs.

Our study has several limitations. It is highly unlikely that the rat’s hematopoietic system is essentially identical to that of the human. In fact, recent studies suggest that in the rat the shift to medullary hematopoiesis is completed several weeks to months after birth, rather than during gestation, as in the human fetus.^36^ In addition, the physiologic mechanisms that result in hypoxia in the human fetus are obviously quite different than the maternal exposure used in our model. Because of these differences, it is difficult to state whether or not our conclusions would be applicable to the human fetus. It is, however, interesting to note that our results are consistent with the observations made by Korst et al. and Phelan et al. who concluded that the highest NRBC counts are found in human fetuses with a prolonged and persistent (from admission to delivery) non-reactive fetal heart rate pattern.^1^,^20^ Our data support the assertion of previous investigators that extremely elevated NRBC counts in the term human newborn may be secondary to prolonged periods of hypoxia.^1^,^10^ In addition, our conclusion that hypoxic exposure of less than 24 hours *did not* result in a significant elevation of fetal NRBCs is somewhat questionable. As stated in Table 1, the differences between study and control fetuses were not statistically meaningful at exposures times of less than 24 hours. However, with *P* values of 0.05 (at 2 and 12 hours of exposure) it is possible that a larger study would conclude that short-term exposure to hypoxia is also associated with elevation of NRBCs, as has been suggested in some human studies.^8^ Regardless of the exact *P* value, our data support suggestions by previous investigators that acute hypoxic events are not likely to result in extreme elevations in NRBC counts over baseline levels.^1^ Our data also demonstrate a wide variability in NRBC count in the normal (control) individual, a finding occasionally noted in normal term human newborns.^26^

The principal strength of our study is derived from the ability tightly to control an animal model so that the only difference between study and control groups is the exposure to severe hypoxia. For obvious reasons, it would be impossible to control a human study to such an extent. This type of model allows us to eliminate the potential influence of other factors that may affect the NRBC count in the human, such as prematurity or infection/inflammation.^33^,^43^

Previous retrospective studies of children with cerebral palsy have suggested that the timing of antenatal hypoxic neurologic injury may be determined with use of NRBC counts.^6^ The use of an animal model has allowed us to address this issue and manipulate the intrauterine environment in a way not possible in humans. Our results clearly indicate that in the rat model, fetal NRBCs are significantly elevated following 24 hours of maternal exposure to severe hypoxia.

## Abbreviations

ICC: intraclass correlation coefficient
NRBC: nucleated red blood cell
WBC: white blood cell
